# Editorial: Endothelial activation and microcirculatory disorders in sepsis and critical illness, volume II

**DOI:** 10.3389/fmed.2024.1477041

**Published:** 2024-08-19

**Authors:** W. Conrad Liles, Jeremie Joffre

**Affiliations:** ^1^Department of Medicine (Primary), University of Washington, Seattle, WA, United States; ^2^Departments of Laboratory Medicine and Pathology, Pharmacology, and Global Health, University of Washington, Seattle, WA, United States; ^3^Sepsis Center of Research Excellence-UW (SCORE-UW), University of Washington, Seattle, WA, United States; ^4^Center for Lung Biology, University of Washington, Seattle, WA, United States; ^5^Medical Intensive Care Unit, Hopital Saint Antoine, Sorbonne University, Assistance Publique-Hopitaux de Paris, Paris, France; ^6^Centre de Recherche Saint-Antoine (CRSA), INSERMUMR_5938, Paris, France

**Keywords:** sepsis, critical illness, endothelium, end-organ injury, microvascular disorder

As co-guest editors for *Frontiers in Medicine*, we are pleased to announce the publication of the second volume (Volume II) of the Research Topic collection, “*Endothelial activation and microcirculatory disorders in sepsis and critical illness.”* This Research Topic collection welcomed submissions focused on the following areas of biomedical research:

1) Basic endothelial biology addressing the role of the endothelium on immune regulation, coagulation/fibrinolysis, oxidative stress, leukocyte/platelet adhesion to the vasculature, rheology, and vasoactivity;
2) Biomarker studies;
3) Translational studies;
4) Endothelial heterogeneity;
5) Assessment and monitoring of microcirculatory disorders in critical illness or major surgery; and
6) Bioinformatic and digital solutions for real-time multiparametric hemodynamic assessment and/or prediction outcome.

As previously highlighted in the editorial introducing Volume I of this Special Topic, the endothelium is one of the largest organs in the human body and lines the blood vessels and microcirculation of all the vital organs (1). As such, it functions as a massive and critical surveillance system to orchestrate recognition and host response to pathogens vial pathogen-associated molecular patterns (PAMPs) and endogenous “alarmins” via danger-associated molecular patterns (DAMPs). Although sepsis research was conventionally focused on deleterious host-mediated inflammatory responses, it is now recognized that endothelial activation/dysfunction affecting vital organs, rather than solely inflammation *per se*, plays a major biological role in the pathophysiology of sepsis and associated clinical outcomes (1).

Despite decades of research, sepsis remains a formidable enigma and a major challenge in biomedical research. Individuals who develop sepsis and related conditions continue to experience adverse clinical outcomes and end-organ injury, including acute kidney injury (AKI), acute lung injury and the clinical syndrome of acute respiratory distress syndrome (ARDS), and multiple organ dysfunction syndrome (MODS). Currently, no specific therapeutic agent is available for the treatment of sepsis. Novel, rational, and effective therapeutic strategies for the treatment of clinical sepsis are a major unmet need in clinical medicine.

This Research Topic is focused on the role and consequences of endothelial activation/dysfunction in the pathogenesis of sepsis and its complications. By elucidating the key mechanism(s) regulating endothelial responses in sepsis, it is Volume II includes 1 comprehensive review, 2 narrative mini-reviews, and 5 original research manuscripts.

In their comprehensive review, Cleuren and Molema provide an overview of the current understanding of the heterogeneity and complexity of organ-specific microvascular endothelial cell responses at the molecular level in sepsis. These insights are based on recent studies that have investigated endothelial responses across organ systems in patients with sepsis and in pre-clinical animal models of experimental sepsis. Cleuren and Molema believe that results from upcoming studies employing single cell RNA sequencing and spatial transcriptomic technologies will be particularly informative. It is anticipated that these studies will significantly increase our knowledge and understanding of distinct sepsis sub-phenotypes, thereby leading to novel and rational precision medicine approaches for the treatment of individuals with sepsis in order to prevent sepsis-related specific organ injury and MODS.

The narrative mini-review by Cusack et al. in Volume II discusses our current understanding of heterogeneity of ARDS, with an emphasis on the mechanistic role of endothelial damage in the pathogenesis of acute lung injury/ARDS. The authors include an overview of potential novel therapeutic strategies that could be employed to decease endothelial activation/dysfunction and, thereby, prevent or limit ARDS. Sublingual videomicroscopy, and its potential deployment at bedside to monitor microcirculatory dysfunction in sepsis is the subject of the mini-review by Damiani et al.. The authors propose that sublingual microvascular monitor could be used to evaluate and monitor resuscitation strategies in patients with sepsis.

Five original research manuscripts are included in Volume II:

1. Ueno et al. report the results of a single-center prospective observational to investigating the effects of continuous renal replacement therapy (CRRT) on circulating cytokine levels study in a cohort of patients who had undergone surgery for an acute abdomen. Hemodynamic parameters were improved in patients that received CRRT employing a polymyxin B immobilized fiber. This improvement in hemodynamics was associated with a reduction in circulating cytokines, including IL-6. The authors suggest that their findings indicate a potential therapeutic role for CRRT in septic shock via modulation of endothelial activation/dysfunction caused by pro-inflammatory cytokines, including IL-6.
2. The glycocalyx is a structure that covers the luminal side of vascular endothelial cells and plays an important role in the maintenance of endothelial barrier integrity and function. Du et al. report the results of a single-center study in China investigating evidence of glycocalyx destruction and shedding in hemorrhagic fever with renal syndrome (HFRS) caused by Hantavirus infection in China eastern Asia, especially in China and Korea. Clinical manifestations of HFRS include fever, shock, hemorrhage, and AKI. The authors found that measured laboratory parameters of glycocalyx destruction and shedding were associated with endothelial dysfunction and microvascular leak in HFRS. These results suggest that exfoliated glycocalyx fragments detected in the peripheral blood may have potential for evaluation of disease severity and clinical prognosis in patients with HFRS.
3. Mid-regional pro-adreomedullin (MR-proADM), an endothelium-related peptide, which has been previously been reported as a prognostic biomarker in respiratory infections and sepsis, was examined in COVID-19 by Montrucchio et al.. In this single-center, prospective study from an academic medical center in Italy, elevated circulating levels of MR-proADM were associated with mortality risk in this critically ill cohort of ICU patients with severe COVID-19.
4. Ortega-Martorell et al. investigated whether sepsis-induced coagulopathy (SIC) was associated with new-onset atrial fibrillation in individuals with critical illness from data extracted from a large Dutch database. Although SIC was associated with the development of atrial fibrillation, older age was the dominant risk factor identified for first episodes of atrial fibrillation in patients admitted in sinus rhythm without SIC.
5. Bokoch et al. examined the effects of serum from with cirrhosis undergoing liver transplantation. They found that these serum samples induced permeability of cultured human pulmonary microvascular cells *ex vivo*. These findings suggest that increased endothelial permeability may contribute mechanistically to organ injury, including acute lung injury, and adverse clinical outcomes during liver transplantation.

The manuscripts published in Volume II complete the special topic collection, “*Endothelial activation and microcirculatory disorders in sepsis and critical illness.”* This Research Topic was collated to address an important area of current research in sepsis and critical illness. The co-guest editors have selected manuscripts that should be of interest to a broad readership in the biomedical research community.
